# Prediction and Diagnosis of Respiratory Disease by Combining Convolutional Neural Network and Bi-directional Long Short-Term Memory Methods

**DOI:** 10.3389/fpubh.2022.881234

**Published:** 2022-05-04

**Authors:** Li Li, Alimu Ayiguli, Qiyun Luan, Boyi Yang, Yilamujiang Subinuer, Hui Gong, Abudureherman Zulipikaer, Jingran Xu, Xuemei Zhong, Jiangtao Ren, Xiaoguang Zou

**Affiliations:** ^1^Department of Respiratory and Critical Care Medicine, First People's Hospital of Kashi, Kashi, China; ^2^Department of Clinical Research Center of Infectious Diseases (Pulmonary Tuberculosis), First People's Hospital of Kashi, Kashi, China; ^3^State Key Laboratory of Pathogenesis, Prevention and Treatment of High Incidence Diseases in Central Asia, Xinjiang Medical University, Ürümqi, China; ^4^Department of Preventive Medicine, School of Public Health, Sun Yat-sen University, Guangzhou, China; ^5^Department of Software, Sun Yat-sen University, Guangzhou, China

**Keywords:** respiratory disease, convolutional neural network, long-short-term memory network, predictive diagnosis, medical records

## Abstract

**Objective:**

Based on the respiratory disease big data platform in southern Xinjiang, we established a model that predicted and diagnosed chronic obstructive pulmonary disease, bronchiectasis, pulmonary embolism and pulmonary tuberculosis, and provided assistance for primary physicians.

**Methods:**

The method combined convolutional neural network (CNN) and long-short-term memory network (LSTM) for prediction and diagnosis of respiratory diseases. We collected the medical records of inpatients in the respiratory department, including: chief complaint, history of present illness, and chest computed tomography. Pre-processing of clinical records with “jieba” word segmentation module, and the Bidirectional Encoder Representation from Transformers (BERT) model was used to perform word vectorization on the text. The partial and total information of the fused feature set was encoded by convolutional layers, while LSTM layers decoded the encoded information.

**Results:**

The precisions of traditional machine-learning, deep-learning methods and our proposed method were 0.6, 0.81, 0.89, and *F*1 scores were 0.6, 0.81, 0.88, respectively.

**Conclusion:**

Compared with traditional machine learning and deep-learning methods that our proposed method had a significantly higher performance, and provided precise identification of respiratory disease.

## Introduction

Respiratory diseases, including pulmonary tuberculosis (PTB), chronic obstructive pulmonary disease (COPD), pulmonary thromboembolism (PTE), and bronchiectasis, are among the most common diseases clinically. These diseases have common symptoms such as cough, sputum expectoration, wheezing, and chest pain, but the treatment and follow-up of each disease are completely different (1–4). The similar symptoms among these diseases make timely diagnosis difficult. Misdiagnosis is common in primary hospitals, and can lead to inappropriate treatment, prolonged recovery time, and potential deterioration, and limited experience of doctors at primary hospitals also worsens the situation (5).

The dry climate, air pollution and rural biofuels have led to a high incidence of chronic airway diseases, and the limited experience and medical equipment of doctors in primary hospitals in Kashi area of China, make it difficult to identify similar diseases (6). Therefore, we established a respiratory system big data platform in Kashi, using machine learning, natural language recognition and extraction methods to discuss the information in patients' electronic medical records, and established algorithm models to achieve high accuracy through model autonomous learning. In practical applications, machine learning methods provide technical support for precision medicine and efficient medicine (7).

Research shows that machine learning has been applied in medical treatment, including diagnosis, recurrence prediction, and medication (8). The purpose of machine learning is performing high precision classification or discrimination of unknown predicted diseases through autonomous learning and data analysis. In a world of ever-growing data where hospitals are slowly adopting big data systems (9), there are major benefits to using data analytics in the healthcare system to provide insights, augment diagnosis, improve outcomes, and reduce costs (10). In particular, successful implementation of machine learning enhances the work of medical experts and improves the efficiency of the healthcare system (11). Significant improvements in diagnostic accuracy have been shown through the performance of machine-learning models along with clinicians (12).

**Figure 1:**
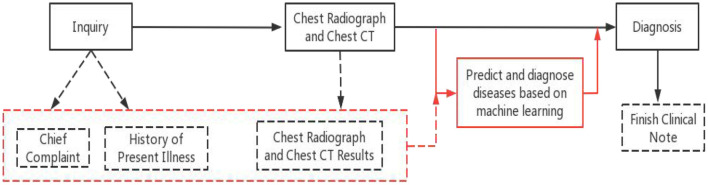
Original diagnostic procedures and our proposed method. Black solid line and boxes are original procedure, black dashed lines and boxes are generated clinical notes at each step. Red solid lines are the additional step of using the proposed method and the red dashed box and line are the existing clinical notes used by the proposed method.

Over the past few years, a large number of clinical studies have used various type of machine learning. Researchers such as Patrício (13) used machine learning algorithms to predict breast cancer in blood sample data compared with traditional methods, and found that machine learning methods greatly shortened the diagnosis time and improved the accuracy. A combined deep learning and multi-level feature extraction methodology (CNN-LSTM) were proposed to identify COVID-19 CT scans and chest X-rays (14). A CNN-LSTM hybrid forecasting model has been proposed, which can precisely foresee the COVID-19 episode across India contrasted with other conventional models. In this study, we discussed the possibility of effectively analyzing electronic medical records without using any manual annotations, and evaluate the performance which an approach based on the fusion of two machine-learning methods for the prediction and diagnosis of respiratory diseases. Comparison of the original diagnostic program and our proposed method is shown in Figure 1.

Our proposed method provided reliable assistance without requiring any changes to the original diagnostic procedure. Despite the known association between diseases, the models could predict these diseases, and benefited a wide range of patients. In turn, we were able to identify the common features between the diseases that affected prediction. The respiratory system big data platform was used to train and test multiple models for the prediction of these diseases.

## Materials and Methods

### Inclusion Criteria

(1) Clinical records from January 2018 to August 2021 were collected from the Respiratory Department of the First People's Hospital of Kashi, which is a grade AAA hospital in the Southern Xinjiang. (2) Clinical records were collected for hospitalized patients diagnosed with COPD, PTB, PTE or bronchiectasis disease (3) Clinical records of patients first hospitalization. (4) Patients with the following clinical records : age, sex, occupation, ethnicity, history of present illness (HPI), chief complaint (CC), imaging examination (chest CT), disease history, smoking history, allergy history, and physical examination results.

### Exclusion Criteria

(1) Patients with any two or more of these diseases at the same time. (2) Patients with lack of clinical records.

### Pre-processing of Clinical Record Texts

**Figure 2:**
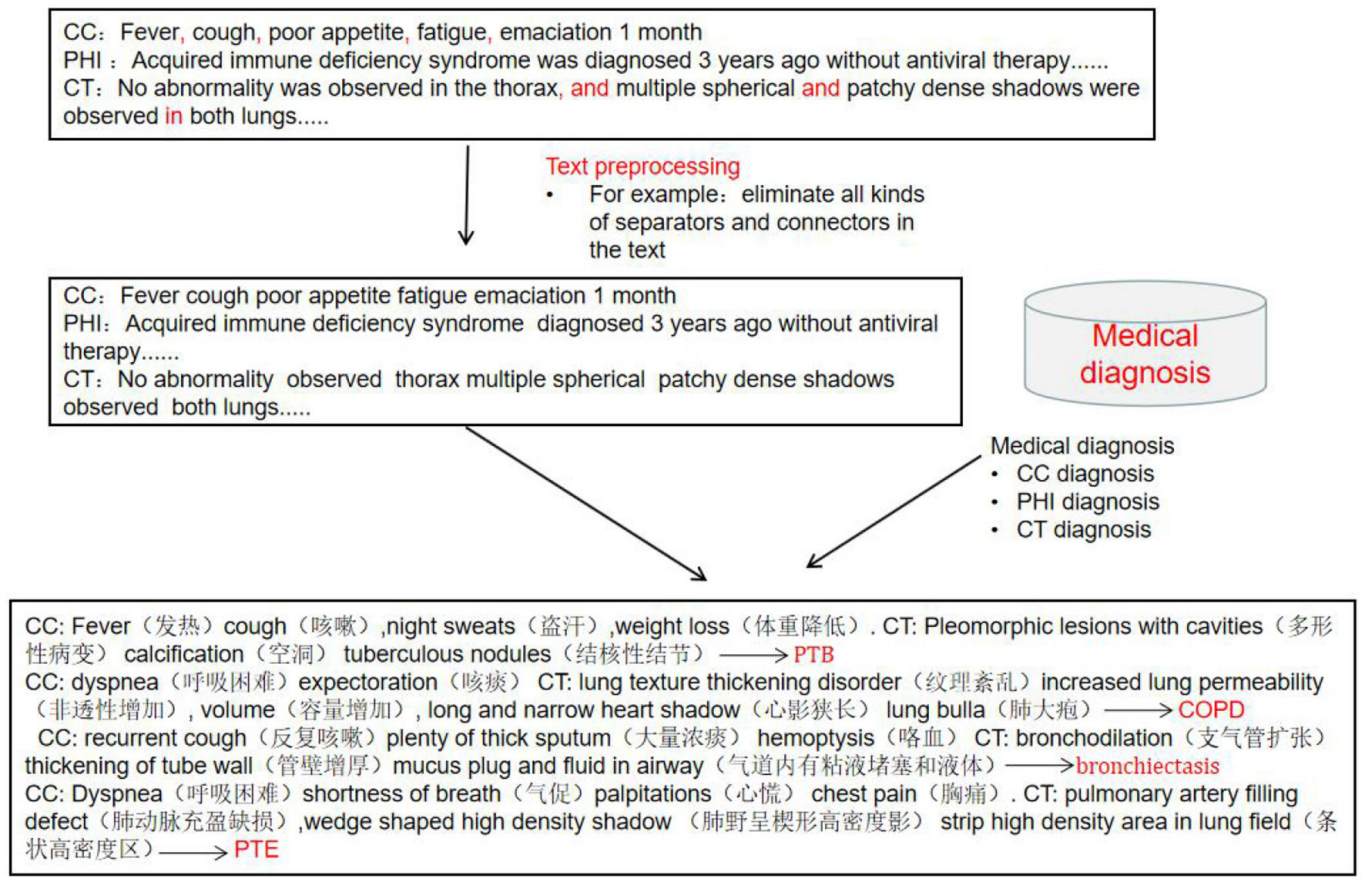
Text pre-processing and medical diagnosis.

The clinical records included chief complaints, history of present illness and CT imaging results. The preprocessing process of clinical record texts is shown in Figure 2. (1) Stop word setting: filter out adverbs, conjunctions, prepositions and modal auxiliary words in the text, and same words that have no actual meaning or have nothing to do with disease diagnosis terms. (2) Special symbol filtering: eliminate all kinds of separators and connectors in the text, such as punctuation, (“,”, “.”, “–”, etc.) and some meaningless separators (“space”, “|”, etc.) and other symbols (“*”, “⋆”, etc.). “?”, “+” and “–” indicate suspicion of disease, positive and negative, and needed to be retained. (3) Word segmentation dictionary settings: Based on Python “jieba” segmentation (15), the Medical Professional Term Dictionary compiled by Tsinghua University was introduced into the module as the word segmentation dictionary, improving the efficiency of model word segmentation process.

### Generate Word Vectors of Text

**Figure 3:**
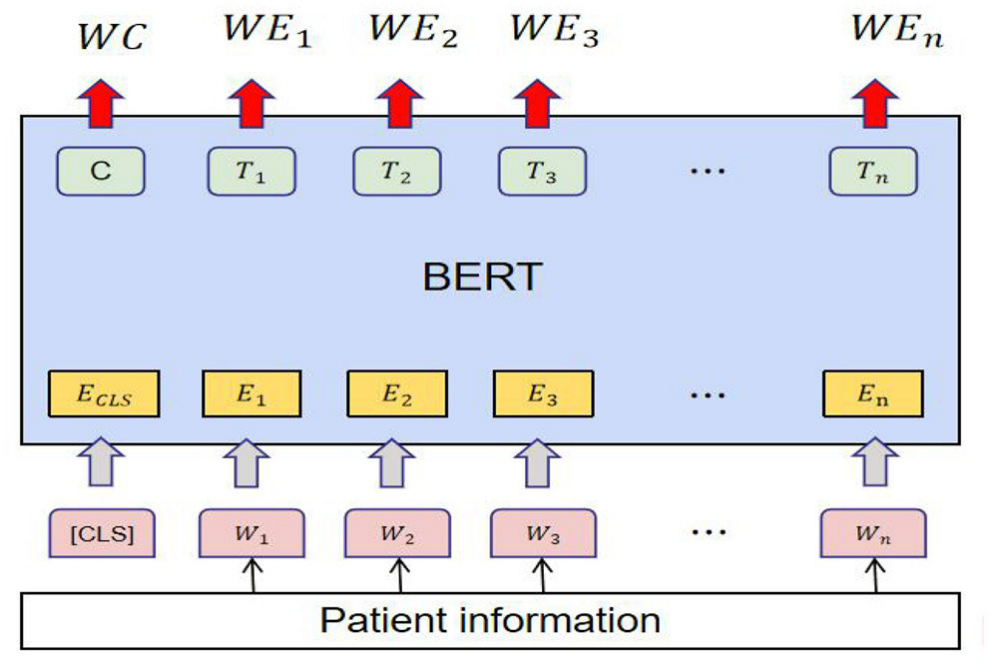
BERT model Generate word vectors of text.

Instead of directly processing text input, the text data were fed into the word embedding to generate an embedding for each word. All texts obtained word vectors through the BERT model (16), and these word vectors were mapped into a high dimensional vector space *V 2* RC using the *word2vec* method. Each sentence was converted into a text embedding *T* = [*t*_1_; *t*_2_; · · ·; *t*_L_] *2* RC × *L*, with each *t*_i_ *2 V*. The text embedding select token embedding, drop segment embedding, and position embedding (17) (Figure 3).

### Feature Extraction

#### TextCNN

**Figure 4:**
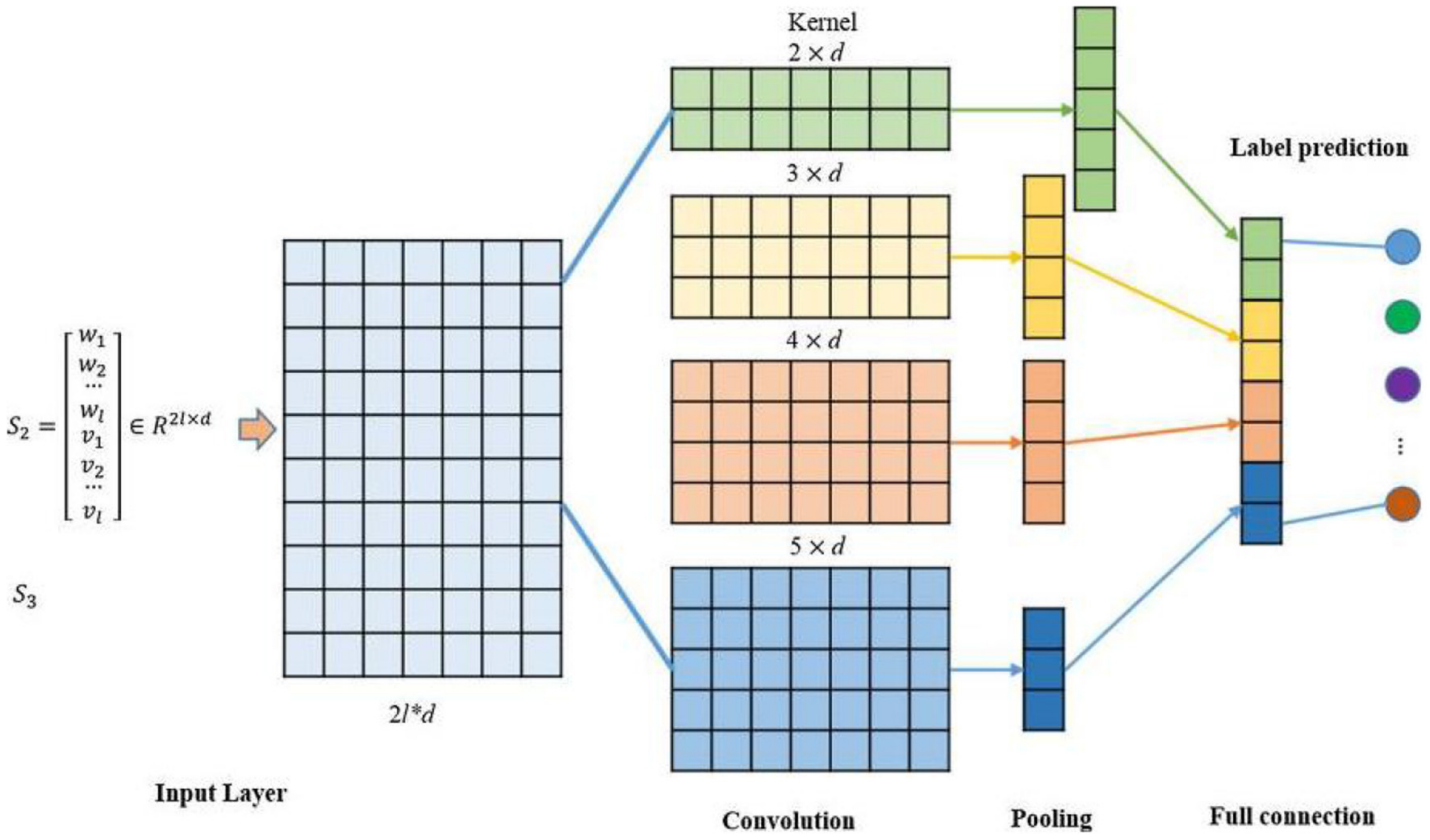
Convolutional neural network.

TextCNN is a variant of convolutional neural network (CNN). TextCNN uses a *k*-dimensional vector to represent a word in a sentence (18). Each word corresponded to a one-dimensional vector, which was classified using CNN. The network model consisted of 200 filters whose window sizes were 2, 3 and 4. The specific model structure is shown in Figure 4. The training model consisted of an embedding layer, convolutional layer, pooling layer, and fully connected layer. If the Eigenmaps were obtained, we pooled them according to the maximum value of each convolution value. Feature extraction was the main function of the convolution and pooling layers (19). It extracted the main features from the text sequences of certain lengths through partial word order information. Then, convolution is used to learn the hierarchical features of words to sentences and sentences to paragraphs (20). PHI information: “The patient coughed and expectorated without obvious cause, white sticky sputum with little amount and intermittent low fever and other symptoms since January,” Firstly, word meaning is extracted, such as “cough,” “sticky sputum,” and raising the sentence meaning to obtain more accurate text feature classification.

#### LSTM

Long-short-term memory network is a variable length neural network. The model has a short-term memory function. It is more suitable for learning text features with long and short memory, and then realizing text feature classification based on this. For example: “1 month ago, intermittent cough and unwell expectoration occurred without obvious reasons. Two days ago, she was admitted to hospital with severe cough and sputum, large sputum volume, not easy to cough, accompanied by symptoms such as shortness of breath and dyspnea.” In order to judge the final result, it is necessary to combine the medical history of 1 month ago and the present, LSTM short and long time memory model with the semantics of two sentences to obtain more accurate text information, and achieve text feature classification. The model consists of input gate *i*, forgetting gate *f*, and output gate *h* (21, 22). The equations are shown:


(1) $$\begin{array}{lll} i_{t} & = & \sigma \left(W_{i}\cdot \left[h_{t-1},x_{t}\right]+b_{i}\right)\;\\ f_{t} & = & \sigma \left(W_{f}\cdot \left[h_{t-1},x_{t}\right]+b_{f}\right)\;\\ \tilde{C_{t}} & = & \tanh\left(W_{c}\cdot \left[h_{t-1},x_{t}\right]+b_{c}\right)\;\\ C_{t} & = & f_{t}\;*\;C_{t-1}+i_{t}\;*\;\tilde{C_{t}}\\ O_{t} & = & \sigma \left(W_{o}\left[h_{t-1},x_{t}\right]+b_{o}\right)\;\\ h_{t} & = & O_{t}\;*\;\tanh\left(C_{t}\right)\; \end{array}$$

*i*_*t*_ is the input gate, while *f*_*t*_ is the forget gate, and *O*_*t*_ is the output gate at moment *t*. $\tilde{C_{t}}$is the input in the neuron at time *t*. *C*_*t*_ is the updated value in the neuron at time *t*. *h*_*t*_ stores the value of the hidden layer at time *t* and before. The value of σ is the activation function sigmoid. *W* and *b* are the weight and bias terms.

### Disease Identification

**Figure 5:**
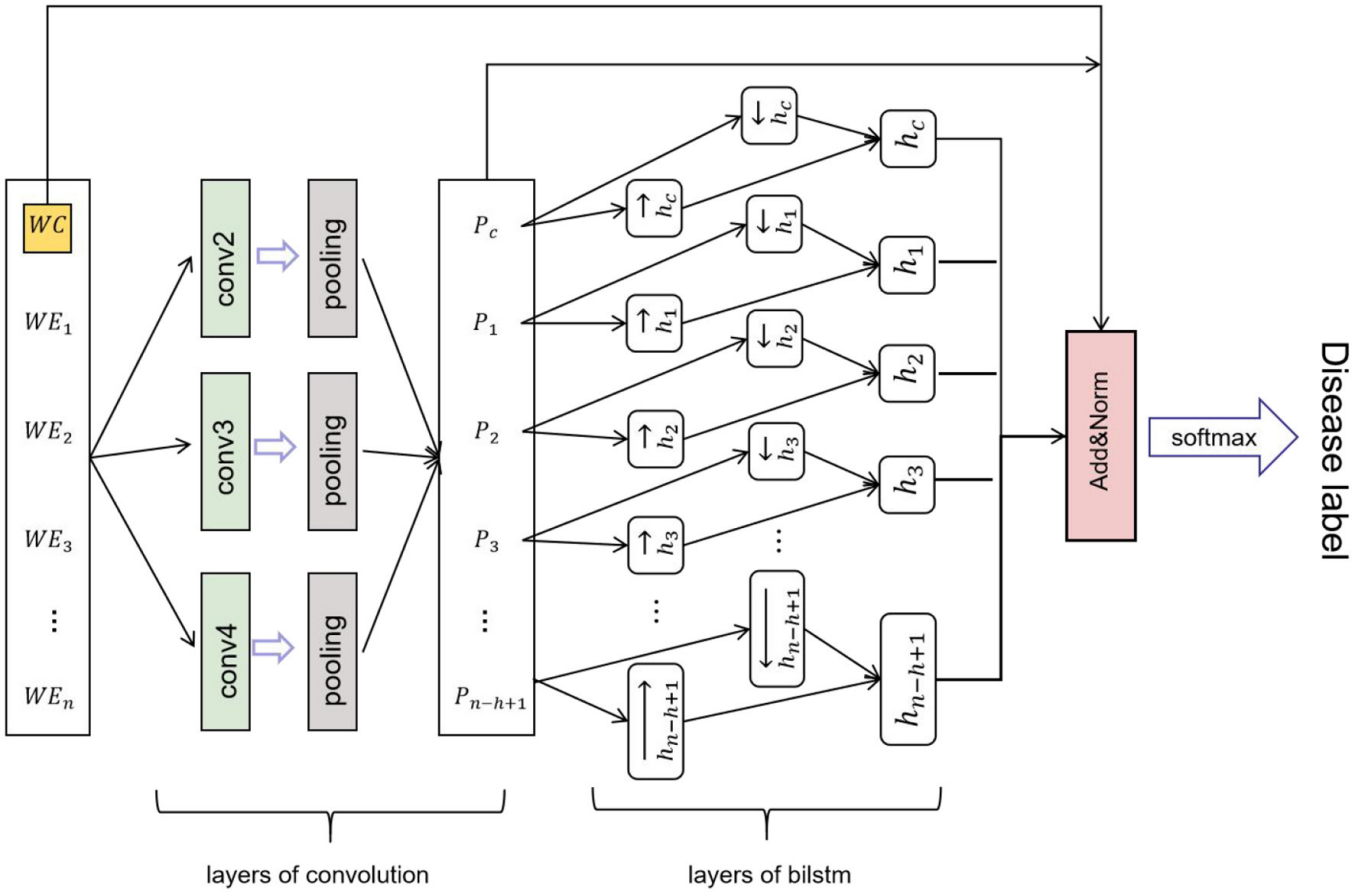
Data feature exaction through CNN-BILSTM model.

**Figure 6:**
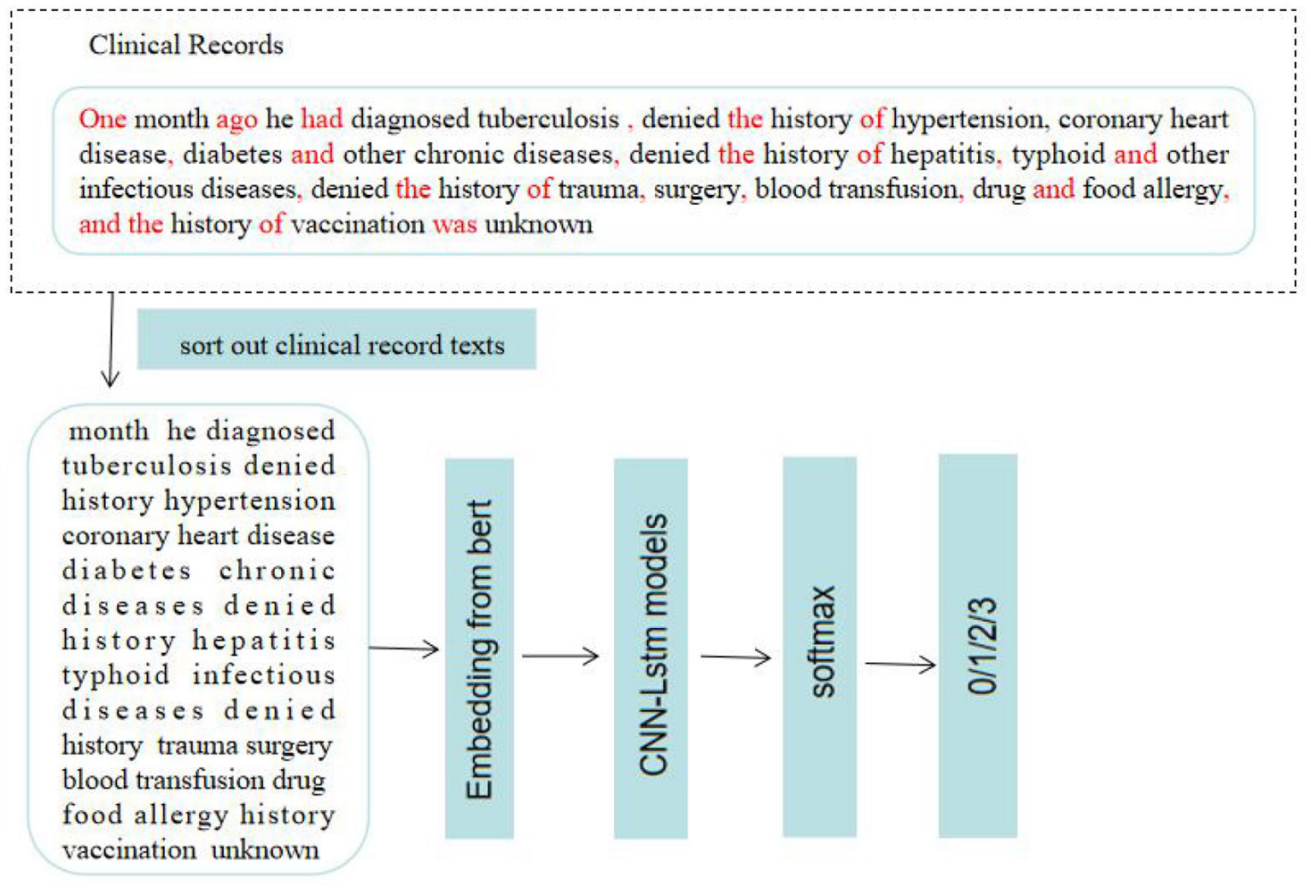
The process of disease identification. Based on machine learning to predict and diagnose respiratory diseases. 0: PTB; 1:COPD; 2: PTE; 3: bronchiectasis.

Convolutional neural network-BILSTM model, which uses a BILSTM layer and a CNN layer to extract data features. BILSTM is a bidirectional LSTM that extracts bidirectional features of text at the same time to obtain better classification results. BILSTM can capture the two-way semantic dependence from front to back and from back to front through two LSTMs in different directions, thereby effectively combining contextual information (23). The features extracted using maximum-pooling layers are often passed to the fully connected layer for classification in CNN networks. However, in the proposed CNN network, the sequence of deep features passed to the LSTM layer rather than directly through the fully connected layer for classification. The CNN network efficiently extracted the text, while the LSTM network detected long-short-term dependencies (24). The CNN-BILSTM model contained two phases (Figure 5). Phase one included convolution layers and maximum-pooling layers, and phase two consisted of the LSTM layer. The partial and total information of the fused feature was encoded by the convolution layers, while the LSTM layer decoded the encoded information. Figure 6 shows the whole process of disease identification.

### Evaluation Matrices

We chose three evaluation matrices such as *F*1 score, precision (*P*) and recall rate (*r*) for each disease to provide a comprehensive evaluation of our proposed method (25). Recall measured the ability to identify positive cases. If disease was defined a positive criterion, recall described the proportion of all real patients identified by the machine-learning method and treated in hospital. Precision was the ratio of all correctly classified medical records to all actually classified medical records. *P* and *r* were defined as follows:


(2) $$\begin{array}{lll} P & = & \;\frac{\text{TP}}{\text{TP}+\text{FP}}\\ r\; & = & \frac{\text{TP}}{\text{TP}+\text{FN}} \end{array}$$

where TP, FN and FP were true positive rate, false negative rate and false positive rate. the *F*1 Score was a comprehensive metric that combined precision and recall. The larger the value, the better the system performance. *F*1 score was defined as follows:


(3) $$\begin{array}{l} F1\;=\frac{2.p.r}{p+r} \end{array}$$

The development and evaluation of the solution were performed in the Python environment (26).

## Results

### Dataset

**Figure 7:**
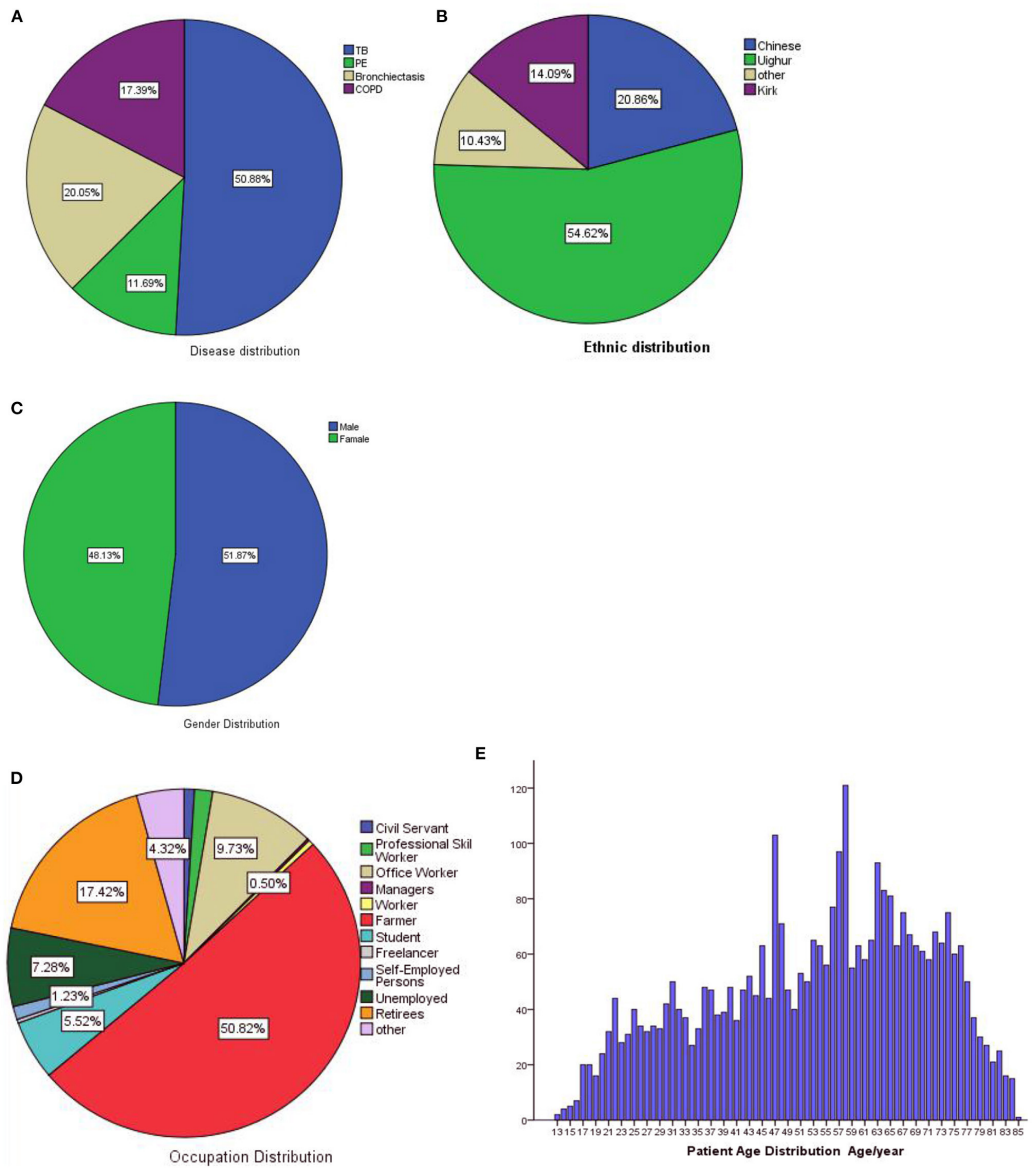
Dataset statistics. **(A)** Disease distribution, **(B)** ethnic distribution, **(C)** gender distribution, **(D)** occupation distribution, **(E)** age distribution.

We collected 3,422 eligible patients, and all clinical records were written in Mandarin. The percentages of each diseases are shown in Figure 7A, there were 1,741 patients with PTB, 400 with PTE, 686 with bronchiectasis, and 595 with COPD. The ethnic distribution is shown in Figure 7B. There are 51.87% patients are male and 48.13% are female, the sex distribution is shown in Figure 7C. The distribution of occupations is shown in Figure 7D, there are 50.82% patients are civil servant, 17.42% are retirees and other. The ages of patients range from 13 years to 85 years old, mean and standard deviation (53.16 ± 17.06), the detailed distribution of patients' age is shown Figure 7E. This study show that the basic information of patients accords with the population distribution in Kashi. Therefore, this model is suitable for the diagnosis and prediction of respiratory diseases in Kashi.

### Model Comparison

To better validate the performance of our proposed method for disease predict and diagnose, we also compared our method with other machine-learning methods. Deep-learning methods improved P from below 0.6 to about 0.81, and our proposed method further improved *P* to 0.89, and *F*1 score: 0.6, 0.81 and 0.88, respectively (Table 1). Our proposed method had a significantly higher performance.

**Table 1:** Performance comparison between the proposed method and multiple benchmark algorithms.

**Method**	***P***	***r***	***F*1 score**
Logistic regression	0.58	0.62	0.60
Decision tree	0.53	0.56	0.54
SVM	0.68	0.65	0.66
CNN	0.85	0.84	0.84
BILSTM	0.77	0.81	0.79
Proposed method (CNN-BILSTM)	**0.89**	**0.87**	**0.88**

### Confusion Matrix Results

**Figure 8:**
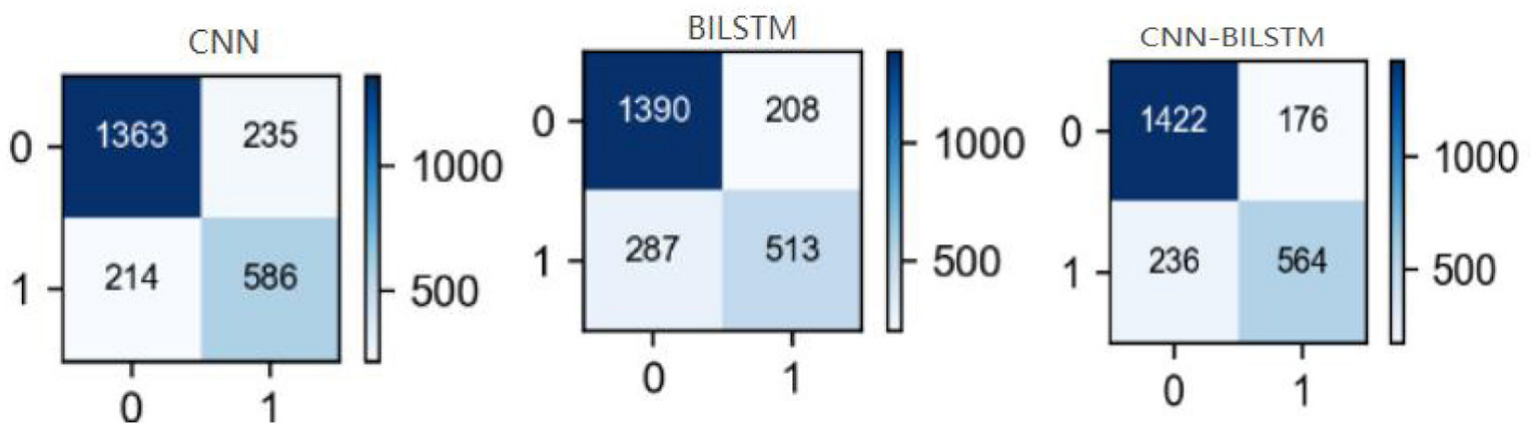
Confusion matrices of each method. Horizontal is predicted value, vertical is actual value, “0” is negative, “1” is positive.

To provide an in-depth understanding of the proposed method's results, we report the confusion matrix of each disease in Figure 8. FP and FN values of CNN, BILSTM and CNN-BILSTM methods were limited to a reasonable range. The FP and FN of the proposed method were lower than those of the CNN and BILSTM methods, while TP was higher.

## Discussion

There are many types of respiratory diseases, and the common symptoms are obviously homogeneous. It is difficult to accurately determine the type of disease based on medical history and physical examination, and misdiagnosis of these diseases leads to inappropriate treatment resulting in prolonged recovery time and potential exacerbation. Performing chest CT examinations on all patients for definite diagnosis may result in a waste of medical resources. Therefore, developing a model for diagnosis of respiratory diseases based on artificial intelligence can achieve diagnosis and prediction of respiratory diseases, and provide diagnostic information for outpatient physicians as a reference, which will help improve the efficiency of medical resource allocation (27).

Traditional machine-learning methods include Logistic Regression (LR), Decision Tree (DT), support vector machine (SVM) (28–30), and various deep learning methods recently used in analysis of clinical records, including the more advanced methods BILSTM and TextCNN. Traditional machine learning methods can not effectively extract features. Some studies explores to build a machine learning model for the differentiation of nontuberculous mycobacteria lung disease (NTM-LD) and pulmonary tuberculosis lung disease (PTB-LD) by using CT images. An artificial neural network (ANN) was used for the prediction of PTB infection (31). Ruihua Guo (32) explored an integrated process to improve TB diagnostics *via* CNNs and localization in chest X-ray (CXR) *via* deep-learning models is proposed, and found that machine learning methods greatly shortened the diagnosis time. Joyce DS (33) to develop machine learning methods to predict COPD using chest radiographs and a CNN trained with near-concurrent pulmonary function test (PFT) data. There is no research on the fusion of two machine learning methods to predict and diagnose respiratory diseases.

This study show, the precisions of traditional machine-learning, deep-learning methods and our proposed method were 0.6, 0.81, 0.89, and *F*1 scores were 0.6, 0.81, 0.88, respectively. CNN-BILSTM method generally outperformed deep-learning models and traditional machine-learning methods in predicting and diagnosing diseases, and usually used for analysis of clinical records, performing high precision classification or discrimination of unknown predicted diseases through autonomous learning and data analysis.

This study had the following limitations. The population was mainly in the Kashi area of Southern Xinjiang, and applicability to the wider population is limited. The epidemiology of different regions differs, so the method needs to be corrected in practical application. Primary healthcare units cannot fully cover the required inspections, such as chest CT. Our dataset consisted of approximately 3,400 clinical records, and further increasing the data set size could improve the performance of our method. Numerical data such as BMI and blood pressure are sparse, and some fields have 90% empty entries, so we excluded numerical data in this study. Extracting numerical values from clinical records more accurately and generating denser data should improve the performance.

## Data Availability Statement

The datasets presented in this study can be found in online repositories. The names of the repository/repositories and accession number(s) can be found at: xjkshospital.com/grxjb.

## Ethics Statement

We confirmed that this study's all methods were carried out in accordance with relevant guidelines and regulations, and all experimental protocols were approved by First People's Hospital of Kashi. Meanwhile, we confirmed that informed consent was obtained from all subjects and/or their legal guardian(s).

## Author Contributions

LL, AA, and QL designed the study, implemented the model, and drafted the manuscript. XZo and JR participated in data pre-processing and manuscript revision and experiment design. BY, XZh, HG, AZ, YS, and JX performed experiments and analyses. All authors have read and approved the final version of this manuscript.

## Funding

This work was supported by the State Key Laboratory of Pathogenesis, Prevention and Treatment of High Incidence Diseases in Central Asia (SKL-HIDCA-2020-KS1). State Key Laboratory of Pathogenesis, Prevention and Treatment of High Incidence Diseases in Central Asia (SKL-HIDCA-2020-10). Tianshan Innovation Team Plan of Autonomous Region (2020D14013).

## Conflict of Interest

The authors declare that the research was conducted in the absence of any commercial or financial relationships that could be construed as a potential conflict of interest.

## Publisher's Note

All claims expressed in this article are solely those of the authors and do not necessarily represent those of their affiliated organizations, or those of the publisher, the editors and the reviewers. Any product that may be evaluated in this article, or claim that may be made by its manufacturer, is not guaranteed or endorsed by the publisher.
